# Ultrasensitive detection of uric acid in serum of patients with gout by a new assay based on Pt@Ag nanoflowers

**DOI:** 10.1039/c9ra06481h

**Published:** 2019-11-11

**Authors:** Xue Wang, Shujun Chen, Xiaomin Tang, Daiqin Lin, Ping Qiu

**Affiliations:** Department of Chemistry, Nanchang University Nanchang 330031 China pingqiu@ncu.edu.cn; The Fourth Affiliated Hospital of Nanchang University Nanchang 330003 China; Jiangxi Province Product Quality Supervision Testing Institute Nanchang 330047 China

## Abstract

A ultrasensitive assay for the determination of uric acid (UA) based on Pt@Ag nanoflowers (Pt@Ag NFs) was constructed. H_2_O_2_ was formed by the reaction of uricase and UA and produced the hydroxyl radical (˙OH). The system was catalyzed by Pt@Ag NFs to change the color of 3,3′,5,5′-tetramethylbenzidine (TMB) from colorless to blue, and the morphology and chemical properties of Pt@Ag NFs were characterized by transmission electron microscopy and X-ray photoelectron spectroscopy. Under the optimized conditions, a linear relationship between the absorbance and UA concentration was in the range of 0.5–150 μM (*R*^2^ = 0.995) with a limit of detection of 0.3 μM (S/N = 3). The method can be applied to detection of UA in actual samples with satisfactory results. The proposed assay was successfully applied to the detection of UA in human serum with recoveries over 96.8%. Thus, these results imply that the UA assay provides an effective tool in fast clinical analysis of gout.

## Abstract 2

A ultrasensitive assay for the determination of uric acid (UA) based on Pt@Ag nanoflowers (Pt@Ag NFs) was constructed.

## Graphical abstract

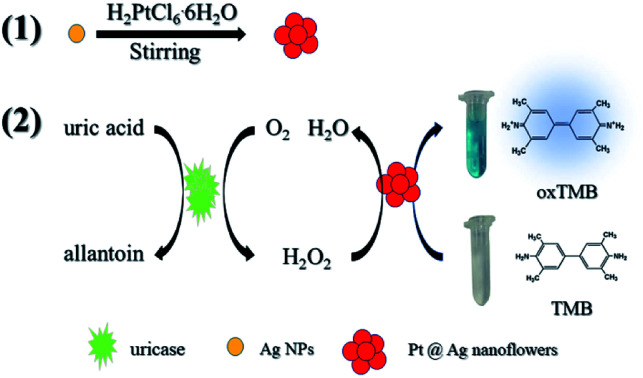

## Introduction

Uric acid (UA) is the final product of human sputum metabolism,^1^ its concentration in serum is 120–480 μM;^2^ many diseases of the human body are related to abnormal uric acid content, such as gout, Lesch-Nyhan syndrome, diabetes, hyperuricemia, heart disease.^3–6^ Monitoring the concentration of UA in human serum is essential and can be used as an early warning for these diseases. Many diseases are caused by diet and lifestyle, so as a remedy, all asymptomatic gout patients should be encouraged to change their lifestyle. This requires the development of a suitable cost-effective monitoring system to provide adequate feedback and treatment guidelines for gout patients.

Gout is an inflammatory reaction that occurs primarily in the joints and forms UA crystals that cause pain.^7^ There is a direct positive correlation between UA levels and the future risk of gout. Specifically, as the concentration of UA increases, the risk of crystal formation increases, thereby increasing the patient's susceptibility to gout.^8^

There are many approaches to detect uric acid, such as, capillary electrophoresis,^9^ fluorometry,^10,11^ chromatography,^12^ electrochemical methods,^13,14^ chemiluminescence^15^ and colorimetry.^16^ Furthermore, the colorimetric method is widely used because of its simple operation, fast analysis speed, high sensitivity in these methods. Currently, there are two main colorimetric methods for the detection of UA. One is a colorimetric assay based on precious metal (*e.g.*, Au and Ag) nanomaterials, in which uric acid can induce nanoparticle aggregation,^17^ etching and anti-etching^18,19^ to cause color changes. However, the dispersion of the nanomaterial is unstable and precipitates easily, which causes the color of the solution to change or even disappear. Many external factors also may cause undesirable changes in the state of nanomaterials,^20^ resulting in unsatisfactory results. The other one is the catalytic oxidation strategy, which bases on the peroxidase-like properties of nanomaterials, such as porous metal–organic framework MIL-53 (Fe),^21^ graphite carbonitride nanosheets^22^ and cobalt selenide nanosheets.^23^ They can change color by H_2_O_2_ catalytic peroxidase substrate. The peroxidase mimic enzymes have the advantages of low cost, good stability, simple preparation, controllable structure and composition, and adjustable catalytic activity. Therefore, this kind of nanomaterials have become an ideal and important colorimetric detection tool.

Precious metal nanomaterials have a wide range of applications in catalysis and assays due to their unique physical and chemical properties.^24,25^ More importantly, the bimetallic nanostructures have synergistic and controllable catalytic properties compared with their mono-metallic nanostructures.^26^ So far, nanomaterials of various shapes have been synthesized by chemical methods, such as nanowires,^27^ nanotubes,^28^ nanorods^29^ and nanowires.^30^ Platinum nanomaterials have attracted much attention due to their excellent catalytic ability and high utilization rate, and have been applied to many fields such as catalysis, battery, sensing and medical treatment.^31–34^ Silver nanoflowers have large specific surface area and good electrical conductivity, especially peroxidase-like properties, which provides an excellent substrate for the construction of the assay.^35–37^ A new type of Pt@Ag nanoflower (Pt@Ag NFs) was prepared by adhesion of platinum nanoparticles with high catalytic activity on the surface of silver nanoflowers to further improve the ability of reducing H_2_O_2_.

This paper presents a method for preparing Pt@Ag NFs using bovine serum albumin as raw material. A colorimetric assay using the peroxidase-like activity based on Pt@Ag NFs was developed to detect UA with sensitivity and high selectivity. Pt@Ag NFs catalyze the colorimetric system of TMB and H_2_O_2_ (which is produced by uricase-specific catalysis of UA), resulting in a solution color changing from colorless to blue. The color change of the solution depends indirectly on the amount of uric acid and can be judged by an ultraviolet-visible spectrophotometer and the naked eye.

## Experimental

### Chemical medicines and instrumentations

Silver nitrate (AgNO_3_), chloroplatinic acid (H_2_PtCl_6_·6H_2_O), bovine serum albumin (BSA), ascorbic acid (AA), 3,3′,5,5′-tetramethylbenzidine (TMB), cysteine (Cys) and glucose (Glu) were purchased in Sinopharm Chemical Reagent Co., Ltd. (Shanghai, China).

Uric acid (UA), uricase, dopamine (DA), and glutathione (GSH) were obtained from Sigma-Aldrich (Shanghai, China). Urea, hydrogen peroxide (30 wt% H_2_O_2_), KNO_3_, Mg (NO_3_)_2_, NaCl, CaCl_2_ were obtained from Beijing Chemical Industry Co., Ltd. (Beijing, China). All reagents are analytical grade and do not require further treatment. Experimental water was ultrapure water (18.25 MΩ, Millipore, USA).

UV-vis spectral measurements were recorded on Agilent 8453 UV-vis spectrophotometer (Agilent Technologies, Santa Clara, CA, USA). The morphology of the Pt@Ag NFs were characterized by transmission electron microscopy (TEM, JEM-2100, JEOL Co., Japan). X-ray photoelectron spectroscopy (XPS) was carried out on EscaLab 250Xi with Al K α X-ray radiation as the source for excitation. Biochemical analysis was measured using biochemical analyzer (AU2007, Beckman, Olympus, USA).

### Preparation of Pt@Ag NFs

The innovative synthesis of Pt@Ag NFs is the key step to this experiment.^37^ Briefly, BSA (5 mg mL^−1^, 20 mL) and AgNO_3_ (10 mM, 10 mL) aqueous solution were mixed and stirred at room temperature for 10 min, then AA (50 mg mL^−1^, 1 mL) was added into the above solution drop by drop. The color of the solution turned light grey, indicating that Ag NFs had been successfully formed. H_2_PtCl_6_·6H_2_O (5.6 mM, 10 mL) was then mixed with the Ag NFs solution and the mixture was stirred at room temperature for a further 10 min until the color turned dark grey. This indicates that Pt@Ag NFs was successfully synthesized, and the obtained product was repeatedly washed three times with water and ethanol, respectively. Finally, the precipitate was re-dissolved in 20 mL of ultrapure water for later use.

### Colorimetric measurement

150 μL of Britton–Robinson buffer (B–R buffer, pH 8.5) was mixed with 50 μL, 100 μg mL^−1^ uricase and 50 μL different concentrations of UA in a 35 °C water bath for 15 min. Then 200 μL, 16 mM TMB, 30 μL Pt@Ag NFs and 1520 μL, 50 mM HAc–NaAc buffer of pH 4.0 were added to the above solution. Shake well to bring the mixed solution into full contact, and incubate for another 40 min in a 55 °C water bath, and collect the data using an UV-vis spectrometer.

### Analysis of UA in human serum

The serum samples of gout patients were collected from the Nanchang University Fourth Affiliated Hospital (Nanchang city, China). The samples treatment was relatively simple. The samples were treated with 4000 rpm centrifugation for 15 min, and the supernatant diluted with phosphate buffer saline (PBS, pH 7.4). The analysis of each serum sample was repeated three times.

## Results and discussion

### Principle of colorimetric assay

**Scheme 1:**
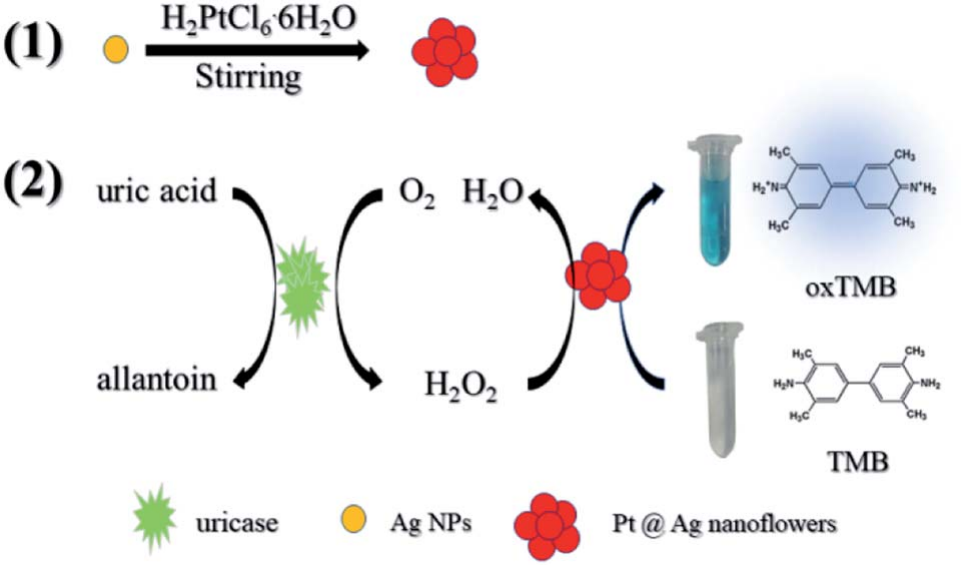
Schematic diagram of the colorimetric detection of UA using H_2_O_2_ and Pt@Ag NFs catalytic oxidation of TMB.

Scheme 1 simply describes the mechanism of the detection of UA by Pt@Ag NFs, which also involves the synthesis of Pt@Ag NFs and the oxidation of uricase. Under weak alkaline conditions, uricase specifically catalyzes UA, producing allantoin, carbon dioxide and H_2_O_2_. Pt@Ag NFs have peroxidase-like activity, which further catalyzes the decomposition of H_2_O_2_ to form hydroxyl radicals (˙OH). ˙OH oxidizes the peroxidase substrate TMB (colorless) to oxidized TMB (oxTMB, blue). These two steps can be expressed by eqn (1) and (2):1
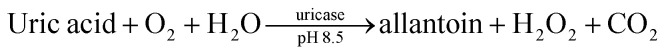
2
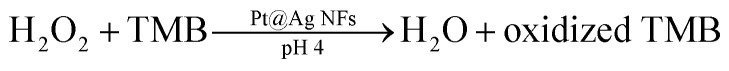


**Fig. 1:**
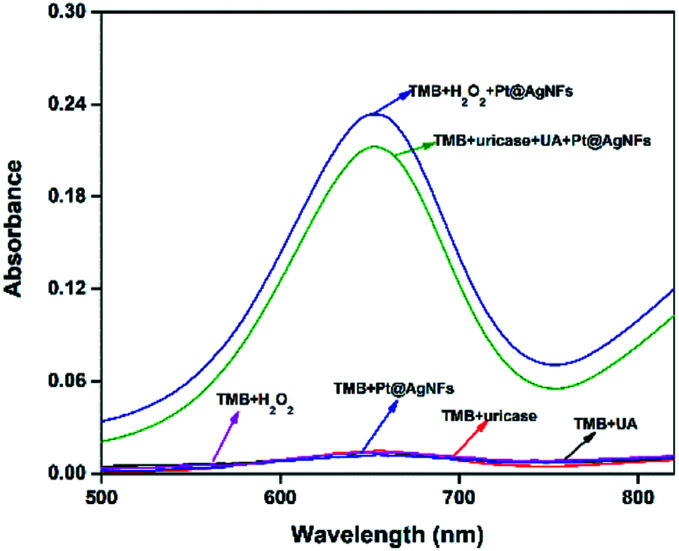
UV-vis spectra of TMB interacting with different substrates. (1) TMB + UA; (2) TMB + uricase; (3) TMB + Pt@Ag NFs; (4) TMB + H_2_O_2_; (5) TMB + UA + uricase + Pt@Ag NFs; (6) TMB + H_2_O_2_ + Pt@Ag NFs; conditions: UA, 20 μM; uricase, 1.25 μg mL; Pt@Ag NFs, 30 μL; TMB, 1.6 mM; H_2_O_2_, 20 μM.

In order to verify the principle of the colorimetric assay, the UV-vis spectra of TMB and the mixture of UA, uricase, Pt@Ag NFs and H_2_O_2_ were compared. As shown in Fig. 1, when TMB was only mixed with one of the above substances, there was no significant absorption peak of oxTMB at 652 nm (black, red, blue and purple curves). In addition, when TMB was added to the mixed solution of Pt@Ag NFs, UA and uricase, the absorbance increased obviously at 652 nm (green curve). At the same time, when H_2_O_2_ was mixed with TMB and Pt@Ag NFs (dark blue curve), a similar phenomenon can be observed, and the absorbance increases. This indicates that Pt@Ag NFs catalyzes H_2_O_2_ and TMB in the same way as UA, uricase and TMB. The results of the whole experiment demonstrated that UA was first catalyzed by uricase to produce H_2_O_2_, and then further catalyzed H_2_O_2_ by Pt@Ag NFs, and the color of reaction substrate TMB changed. Therefore, it can be seen that there is a significant color change from colorless to blue in Scheme 1.

### Structure and properties of Pt@Ag NFs nanoflower

**Fig. 2:**
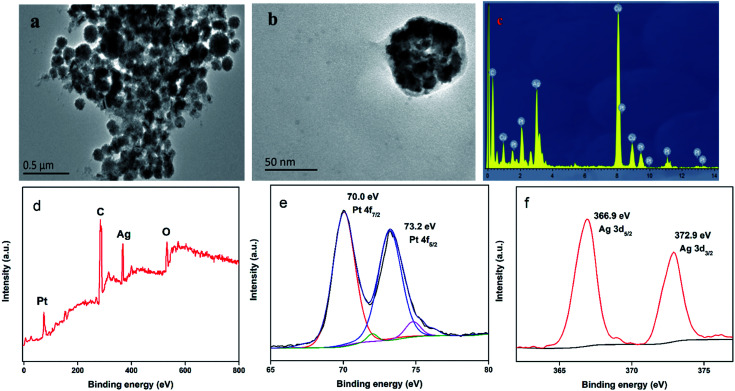
Characterization of the Pt@Ag NFs: (a) TEM image; (b) magnified images; (c) energy dispersive spectra; (d) the XPS spectrum of the Pt@Ag NFs, (e) the high resolution Pt 4f; (f) the high resolution Ag 3d, respectively.

Pt@Ag NFs play a crucial role in the detection of colorimetric uric acid assays. The structure and morphology of Pt@Ag NFs were studied by transmission electron microscopy. Fig. 2a shows the overall morphology of Pt@Ag NFs. The product consists of a large number of flower-like structures with an average diameter of 50 nm, which are well dispersed in water. The magnified TEM image in Fig. 2b shows that the flower-like Pt@Ag nanoparticles are surrounded by a thin layer of BSA. Energy dispersive spectroscopy (EDS) is a method for characterizing elemental composition and X-ray photoelectron spectroscopy (XPS) was performed to confirm the elemental composition and oxidation state of the prepared nanocomposite.^38–40^Fig. 2c shows the EDS spectrum of Pt@Ag NFs, confirming the presence of Pt and Ag in Pt@Ag NFs. The oxidation states and formations of Pt@Ag NFs were determined using XPS measurements. A survey XPS spectrum confirm the coexistence of Pt and Ag elements in Pt@Ag NFs (Fig. 2d). The oxidation states of Pt and Ag are obtained by fitting the peaks in high resolution Pt 4f and Ag 3d XPS spectra (Fig. 2e and f), where the peaks at 70.0, 73.2, 366.9, and 372.9 eV correspond to Pt 4f_7/2_, Pt 4f_5/2_, Ag 3d_5/2_, and Ag 3d_3/2_, respectively, verifying the formation of Pt and Ag.

### Optimization of sensing system

**Fig. 3:**
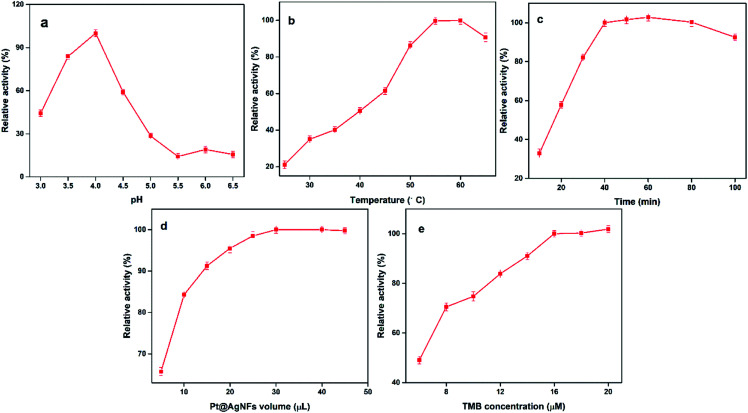
Effects the peroxidase – like activity of Pt@Ag NFs (a) pH, (b) incubation temperature, (c) incubation time (d) Pt@Ag NFs volume and (e) TMB concentration.

It is known from previous literature that the optimal pH, incubation temperature and incubation time for uricase-catalyzed UA reaction are 8.5, 35 °C and 15 min, respectively. Relative activity was used to judge the optimal value of reaction parameters, which can be represented by (*A* − *A*_0_)/*A* (*A* is the absorbance in the presence of UA, *A*_0_ is in the absence of UA). Next, the catalytic activity of Pt@Ag NFs catalyst was optimized to obtain the best sensing response of UA assay. These parameters include pH, incubation temperature, incubation time, Pt@Ag NFs volume and TMB concentration. As can be seen from Fig. 3a, with the increase of pH (3.0–6.5), the relative activity at 652 nm increases firstly and then decreases. At pH = 4.0, the relative activity is the maximum. The catalytic activity of nanozymes at 25–65 °C was studied (Fig. 3b). With the increase of temperature, the intensity of absorption peak at 652 nm gradually increased to the stage of platform, and the incubation temperature was easy to control, which was convenient for practical operation. Fig. 3c shows the effect of incubation time (10–100 min) on the catalytic activity of Pt@Ag NFs. It can be seen from the figure that when the time is 40 min, the relative activity tends to be maximum and there is a small fluctuation. We choose 40 min as the optimum time. At the same time, the volume of Pt@Ag NFs and the concentration of TMB also have a significant effect on the response of the assay. A series of Pt@Ag NFs volumes for UA detection were optimized (Fig. 3d). The relative activity increased gradually at the volume of 10–30 μL of Pt@Ag NFs, but after 30 μL, it decreased. Therefore, 30 μL was chosen as the optimal volume of Pt@Ag NFs. Fig. 3e shows that the relative activity increases with the increase of TMB concentration until saturation. It is worth noting that when the concentration of TMB is more than 1.6 mM, the reaction begins to recrystallize due to its low solubility in the aqueous phase. Therefore, the optimal concentration of TMB was 1.6 mM in the following experiments.

### Linearity and detection limit

**Fig. 4:**
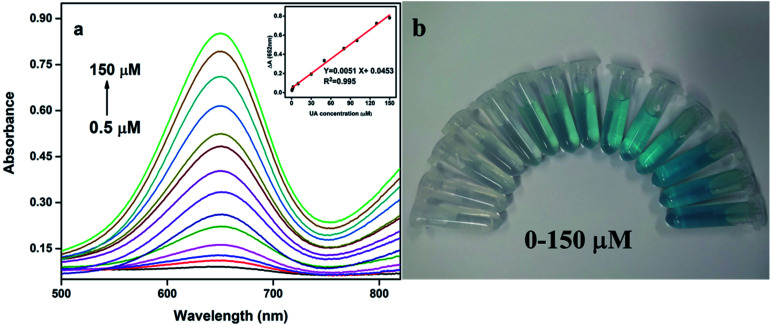
(a) Pt@Ag NFs determination of UA (from low to high: 0.5, 1.5, 2.5, 10, 20, 30, 40, 50, 70, 80, 100, 120, 130, 150 μM), illustrations: linear standard curve of UA; (b) a digital image of reaction systems with different amounts of UA (from left to right: 0, 1.5, 2.5, 10, 20, 30, 40, 50, 70, 80, 100, 120, 130, 150 μM).

In the analysis and detection, the sensitivity of the biological enzyme assay is one of the most important factors. In order to verify the sensitivity of Pt@Ag NFs assay for detecting UA, a series of concentrations of UA standard solutions were detected under optimal conditions. With the increase of UA concentration (0.5–150 μM), the absorbance of the solution at 652 nm also increases (Fig. 4a). Moreover, as shown in Fig. 4b, the color of the solution in the centrifuge tube from left to right also showed a visible change (colorless to blue). The illustration of Fig. 4a is a calibration curve for UA concentration and absorbance. There is a linear relationship between Δ*A* and UA concentration (0.5–150 μM) and the limit of detection (LOD) was 0.3 μM (S/N = 3). The corresponding linear equation is expressed as: Δ*A* = 0.0051*C* + 0.0453 (μM) and the correlation coefficient (*R*^2^) is 0.995. For comparison, Table 1 summarized the analytical parameters of some systems used to detect uric acid. As can be seen from the table, the method used herein has higher sensitivity and lower detection limit.

**Table 1:** Comparison of analytical performances of different UA assays

Assay system	Signal output	Linear range (μM)	LOD (μM)	Real sample	Ref.
TCPO–H_2_O_2_–rubrene	Chemiluminescence	10–1000	5.0	Serum	41
CNCo	Current	2–110	0.83	Serum	42
PrGOa/PB	Current	40–415	8.0	Serum	43
TMB/g-C_3_N_4_/uricase	Colorimetry	10–100	8.9	NR	22
Ag nanoprism/uricase	Colorimetry	1–40	0.7	Serum	16
MPADs	Colorimetry	100–1000	37	Serum	44
N, Fe-CDs	Fluorescence	0.8–133	0.49	Urine	45
Au/Ag NCs	Fluorescence	5–50	5.1	Serum	46
TMB-Pt@Ag NFs/uricase	Colorimetry	0.5–150	0.3	Serum	This work

### Test of selectivity

**Fig. 5:**
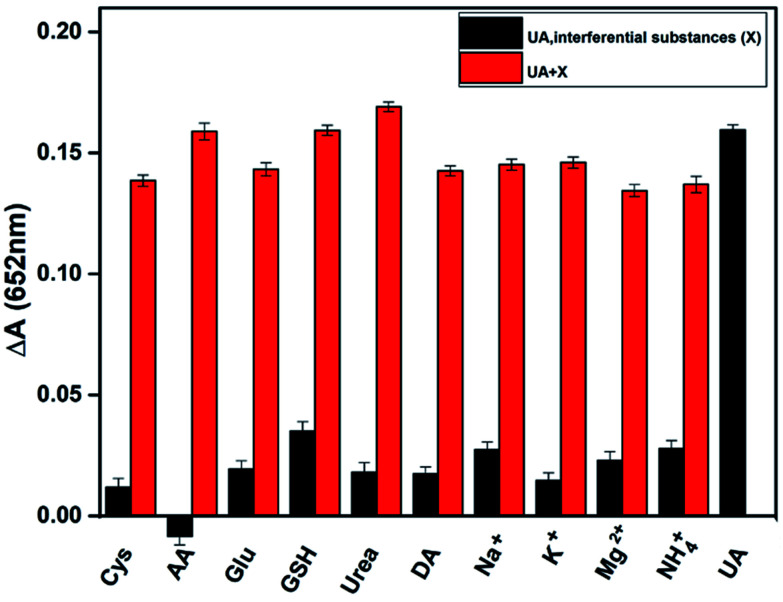
Selectivity of UA assay. In the absence of UA (black) and presence (red), the interfering substances were added to the reaction solution, respectively. The concentration of UA, ascorbic acid, glutathione and dopamine: 20 μM, and the concentration of other interfering substances: 200 μM.

To verify the selectivity of the assay in detecting uric acid, we tested several potential interfering substances in human serum, including cysteine, ascorbic acid, glucose, and several inorganic ions. Under the optimum conditions, the following experiments have been carried out. One is the signal of only UA and interfering substances mixed the detection system (Fig. 5 black column). Compared with the absorbance of adding UA, the interference is basically negligible, which proves the specific catalysis of UA by uricase and the assay has a higher selectivity. The other is the response of uric acid, uricase and various interfering substances coexist (Fig. 5 red column). These results indicate that the assay has a special response to UA regardless of the presence or absence of interfering substances.

### Real sample detection

In order to study the feasibility of UA assay, we used the proposed method and biochemistry analyzer to test the serum samples of 5 gout patients. The serum sample was centrifuged at 4000 rpm for 3 min. Then, 5.6 μL serum mixed with uricase, 4-aminoantipyrine (4-AAP), and 3,5-diphenylamine disodium salt (MADB). And the samples were put in the biochemical analyzer and measure at 660/800 nm. The measurement values of the two methods are almost identical (Table 2), and the average relative error is 3.2%, which means that the developed assay has good accuracy and can be used for the determination of UA in human serum samples. As shown in Table 2, the recoveries of this method are in the range of 96.8–103.3%, and the relative standard deviation (RSD, *n* = 3) of 5 samples is less than 2.6%. These results show that colorimetric assay for the detection of UA has great practical value in clinical application.

**Table 2:** Determination of UA in human serum of goat patients by proposed method and biochemical analysis

Sample	Proposed assay (μM)	Biochemistry analyzer (μM)	Average relative error (%)	Added (μM)	Found (μM)	Recovery (%)	RSD (%)
Serum 1	711	751	3.2	500	1229	103.3	1.6
Serum 2	616	604		500	1122	101.2	1.8
Serum 3	936	967		500	1420	96.8	2.3
Serum 4	522	502		500	1016	98.8	1.9
Serum 5	482	490		500	969	97.4	2.6

## Conclusions

In conclusion, a new assay based on Pt@Ag NFs was constructed to detect UA by the simple colorimetric method. UV-vis spectroscopy showed that Pt@Ag NFs had high sensitivity with the limit of detection for 0.3 μM and high selectivity in the detection of UA. The proposed assay was successfully applied to the detection of UA in human serum with the recoveries in the range of 96.8–103.3%, which has the potential of the simple and fast UA detection platform for point-of-care diagnostics.

## Compliance with ethical standards

The study was approved by the ethics committee of Jiangxi Medical College, and their guidelines were followed throughout this study. The serum samples involved in our research were from gout individuals. Informed consent was obtained from all human participants.

## Conflicts of interest

The authors declare that they have no competing interests.

## Supplementary Material
